# The complete mitochondrial genome sequence of *Fragaria orientalis* (Rosaceae)

**DOI:** 10.1080/23802359.2021.1920498

**Published:** 2021-06-14

**Authors:** Hua-Bo Liu, Jian Sun, Rui Sun, Shuang-Tao Li, Chuan-Fei Zhong, Jing Dong, Lin-Lin Chang, Gui-Xia Wang, Hong-Li Zhang, Yong-Qing Wei, Yun-Tao Zhang, Yong-Shun Gao

**Affiliations:** a Beijing Academy of Forestry and Pomology Sciences, Beijing, P.R. China; b Beijing Engineering Research Center for Strawberry, Beijing, P.R. China; c Key Laboratory of Biology and Genetic Improvement of Horticultural Crops (North China), Ministry of Agriculture and Rural Affairs, Beijing, P.R. China

**Keywords:** *Fragaria orientalis*, mitochondrial genome, phylogenetic analysis

## Abstract

*Fragaria orientalis* Lozinsk. is valuable germplasm material for cross breeding in *Fragaria*. In this study, we assembled the complete mitochondrial genome of *F. orientalis* using a combination of Illumina data and Nanopore data. The mitochondrial genome was 275,143 bp in length, including 29 protein-coding genes, 20 tRNA genes, and three rRNA genes, with a total GC content 45.23%. Seven protein-coding genes contained introns, and three were trans-spliced. Phylogenetic analysis indicated that *F. orientalis* is making a sister clade to the Amygdaloideae species. The complete mitochondrial genome of *F. orientalis* reported in this study will improve our understanding of *Fragaria* evolution.

China is among the most diverse centers of wild strawberry resources, with 14 wild species, including 9 diploid species and all 5 tetraploid species (Sun et al. 2021). *Fragaria orientalis* is a wild tetraploid strawberry, that is distributed mainly in Heilongjiang, Jilin and Liaoning Provinces of Northeast China (The CAS Editorial Committee of the Flora of China 1985; Lei et al. 2017). *F. orientalis* is potentially valuable as breeding material for its aromatic fruit and resistance to low temperatures (Lei et al. 2017). In recent years, breeders have performed interspecific hybridization using *F. orientalis* to improve cold resistance in *Fragaria* (Luo et al. 2018). In this study, we report the complete mitochondrial genome of *F. orientalis* for the first time.

Plant materials were harvested from the China National Strawberry Germplasm Repository (Beijing) (39.9°N, 116.2°E). The specimen and DNA were deposited at the Beijing Academy of Forestry and Pomology Sciences (Yong-Shun Gao, yongshungao@163.com) under the voucher number DF2020-23. Mitochondrial DNA was extracted from fresh leaves using a plant mitochondrial extraction kit (Balb Co., Ltd, Beijing, China) and then sequenced on Illumina NovaSeq 6000 platform and Nanopore platform, respectively. Approximately 4.8 Gb of raw data were generated with insert-size 450 bp paired-end read lengths on the Illumina sequencing platform. A total of 59 Mb long-reads data comprising 23,812 reads were generated on the Nanopore sequencing platform. The mitochondrial genome was *de novo* assembled using the Illumina data by GetOrganelle v1.64 (Jin et al. 2020), and then the assembled scaffolds were aligned against the Nanopore data. All aligned Nanopore reads were extracted and reassembled with the Illumina data by SPAdes v3.13.1 (Antipov et al. 2016). Genome annotation was performed by homologous comparison using BLAST + 2.7.1 (Camacho et al. 2008). The tRNA genes were further annotated using tRNAscan-SE v2.07 (Lowe and Eddy 1997). The annotated mitochondrial genome has been deposited into GenBank under accession number MW464867. The genome sequences were aligned using MAFFT v7.471 (Katoh et al. 2019). Phylogenetic analysis were performed by the maximum likelihood method using RAxML v8.2.12 (Stamatakis 2014) based on GTRGAMMA model with 1,000 bootstrap replicates.

The *F. orientalis* mitochondrial genome was assembled into a single circular-mapping molecule of 275,143 bp, with a GC content of 45.23%. The mitochondrial genome contained a total of 52 genes, including 29 protein-coding genes, 20 tRNA genes, and three rRNA genes. Among the 29 protein-coding genes, seven genes (*nad1*, *nad2*, *nad4*, *nad5*, *nad7*, *ccmFc* and *rps3*) contained introns, and three genes (*nad1*, *nad2*, *nad5*) were trans-spliced. Three genes (*atp6*, *nad4L* and *rps1*) used ACG as the start codon, whereas *nad1* and *mttB* used GTG and TTG, respectively. As the stop codon, fifteen genes ended with TAA, seven genes (*atp4*, *ccmB*, *ccmFn*, *cob*, *cox3*, *nad4* and *rps13*) with TGA, and seven genes (*atp6*, *ccmC*, *matR*, *mttB*, *nad7*, *rps3* and *sdh4*) with TAG. Sequences of 12 common protein-coding genes (*atp1*, *atp9*, *cox1*, *cox2*, *matR*, *nad2*, *nad3*, *nad4*, *nad4L*, *nad6*, *nad7* and *nad9*) were aligned with homologous genes from 10 species. Phylogenetic analysis showed that *F. orientalis* is making an isolated clade, which is a sister to the clade making by species from Amygdaloideae (Figure 1). The complete mitochondrial genome of *F. orientalis* reported in this study will improve our understanding of *Fragaria* evolution.

**Figure 1.**
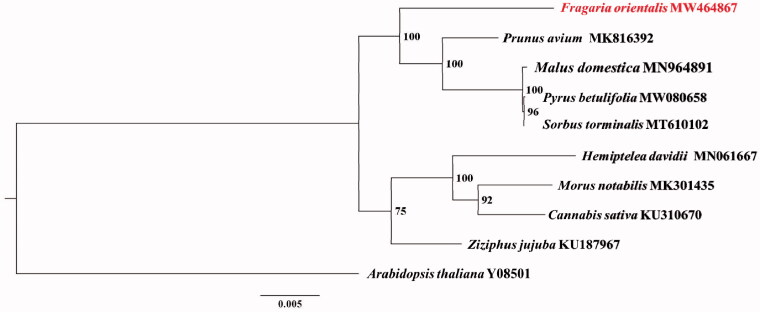
The phylogenetic tree of 10 plant mitochondrial genomes based on 12 common protein-coding genes using *Arabidopsis thaliana* as an out-group. The number on each node indicates the bootstrap value.

## Data Availability

The sequence data that support the findings of this study are openly available in GenBank of NCBI at (https://www.ncbi.nlm.nih.gov/) under the accession no. MW464867. The associated BioProject, SRA, and Bio-Sample numbers are PRJNA693900, SRR13501403, SRR13501404, and SAMN17487953, respectively.
